# Differential expression of epigenetic modifiers in early and late cardiotoxic heart failure reveals DNA methylation as a key regulator of cardiotoxicity

**DOI:** 10.3389/fcvm.2023.884174

**Published:** 2023-03-09

**Authors:** Emma L. Robinson, Pietro Ameri, Leen Delrue, Marc Vanderheyden, Jozef Bartunek, Paola Altieri, Stephane Heymans, Ward A. Heggermont

**Affiliations:** ^1^ Cardiovascular Research Institute Maastricht, Maastricht University, Maastricht, Netherlands; ^2^ Division of Cardiology, Department of Medicine, University of Colorado Anschutz Medical Campus, Aurora, CO, United States; ^3^ Department of Internal Medicine, University of Genova, Genova, Italy; ^4^ Cardiothoracic and Vascular Department, Ospedale Policlinico San Martino – IRCCS Italian Cardiovascular Network, Genova, Italy; ^5^ Cardiovascular Research Center Aalst, Onze-Lieve-Vrouw Hospital Aalst, Aalst, Belgium

**Keywords:** chemotherapy-induced heart failure, anthracycline cardiotoxicity, cardio-oncology, epigenetic memory, DNA methylation, DNA demethylation

## Abstract

**Background:**

Anthracycline-induced cardiotoxicity is a well-known serious clinical entity. However, detailed mechanistic insights on how short-term administration leads to late and long-lasting cardiotoxicity, are still largely undiscovered. We hypothesize that chemotherapy provokes a memory effect at the level of epigenomic DNA modifications which subsequently lead to cardiotoxicity even years after cessation of chemotherapy.

**Methods:**

We explored the temporal evolution of epigenetic modifiers in early and late cardiotoxicity due to anthracyclines by means of RNA-sequencing of human endomyocardial left ventricular biopsies and mass spectrometry of genomic DNA. Based on these findings, validation of differentially regulated genes was obtained by performing RT-qPCR. Finally, a proof-of-concept *in vitro* mechanistic study was performed to dissect some of the mechanistic aspects of epigenetic memory in anthracycline-induced cardiotoxicity.

**Results:**

Correlation of gene expression between late and early onset cardiotoxicity revealed an *R*^2^ value of 0.98, demonstrating a total of 369 differentially expressed genes (DEGs, FDR < 0.05). of which 72% (*n* = 266) were upregulated, and 28% of genes, (*n* = 103) downregulated in later as compared to earlier onset cardiotoxicity. Gene ontology analysis showed significant enrichment of genes involved in methyl-CpG DNA binding, chromatin remodeling and regulation of transcription and positive regulation of apoptosis. Differential mRNA expression of genes involved in DNA methylation metabolism were confirmed by RT-qPCR in endomyocardial biopsies. In a larger biopsy cohort, it was shown that Tet2 was more abundantly expressed in cardiotoxicity biopsies vs. control biopsies and vs. non-ischemic cardiomyopathy patients. Moreover, an *in vitro* study was performed: following short-term doxorubicin treatment, H9c2 cells were cultured and passaged once they reached a confluency of 70%–80%. When compared to vehicle-only treated cells, in doxorubicin-treated cells, three weeks after short term treatment, *Nppa, Nppb, Tet1/2* and other genes involved in active DNA demethylation were markedly upregulated. These alterations coincided with a loss of DNA methylation and a gain in hydroxymethylation, reflecting the epigenetic changes seen in the endomyocardial biopsies.

**Conclusions:**

Short-term administration of anthracyclines provokes long-lasting epigenetic modifications in cardiomyocytes both *in vivo* and *in vitro*, which explain in part the time lapse between the use of chemotherapy and the development of cardiotoxicity and, eventually, heart failure.

## Introduction

For decades, anthracycline-induced cardiotoxicity has been known as a clinical entity (1). Anthracyclines are widely used, potent, and broad-spectrum anti-neoplastic agents, constitute part of standard chemotherapeutical schemas and are lifesaving in hematological and breast cancer. Their utility as an effective anti-cancer therapy must be off-set by the fact that they cause dose-dependent cardiotoxicity (1). Over half of patients with anthracycline exposure have been reported to have some cardiac abnormalities on echocardiogram or gated nuclear angiography by 20 years after diagnosis (1–3). In the worst assessments of rates of anthracycline-induced cardiotoxicity, cancer survivors have a 15-fold increased risk of developing heart failure (HF) in later life and have a 5% increase in likelihood of having a heart transplantation (1, 3). However, in current daily clinical practice, likelihood ratios are lower in part since lower cumulative doses of chemotherapy are used nowadays (4).

Two clinical effects induced by anthracyclines can be distinguished: an acute cardiotoxic response occurring within months or years of anthracycline exposure, and a delayed onset cardiomyopathy (CMP) even several years after chemotherapy cessation (3). Although evolving in a favorable way, present understanding of mechanisms behind cardiotoxic effects of neoplastic treatment is remarkably limited. The suitability of classic circulating diagnostic biomarkers for HF e.g., NT-pro-BNP are largely inadequate (4). Validated prospective tools to identify patients that will develop acute and delayed CMP following anti-cancer therapy are currently non-existent. Preventive measures, let al.one causal treatments, are scarce (2).

Even though anthracycline-induced HF is documented abundantly, and a two-phased pathophysiological mechanism has been put forward (5), in-depth mechanistic insights on how a relatively short-termed administration of chemotherapy can lead to late and long-lasting cardiotoxicity, are still unknown. Since an important proportion of these patients experience late cardiotoxicity, defined as cardiotoxic effects that are identified one and up to 20 years after the administration of potentially cardiotoxic drugs (1, 4), we hypothesized that chemotherapy provokes a long-lasting “memory effect” at the level of non-genetic DNA modifications (“epigenetics”), which can lead to cardiotoxicity even years after cessation of chemotherapy.

Epigenetic mechanisms are an established and accepted means through which pathological cardiac remodeling occurs (6–8). Therefore, in our study we explored the behavior and temporal evolution of epigenetic mechanisms in early and late cardiotoxicity due to anthracyclines by means of deep RNA-sequencing of human endomyocardial biopsies and mass spectrometry of genomic DNA. Based on these findings, validation of differentially regulated genes was obtained by performing RT-qPCR. Finally, a proof-of-concept *in vitro* mechanistic study was performed to dissect some of the aspects of epigenetic memory in anthracycline-induced cardiotoxicity. These findings were the basis for the construction of our hypothetical model of chemotherapy-induced cardiotoxicity based on long-term changes in the cardiac epigenome, or epigenetic memory.

## Materials and methods

### Ethics

Patients provided written informed consent for the procurement of left ventricular endomyocardial biopsy (LV EMB), after dedicated oral and written explanation of the purpose of the procedure by a qualified physician. The Local Medical Ethics Committee (OLV Aalst, Belgium) provided formal approval. The procurement of the LV EMBs respected the principles of the Declaration of Helsinki, locoregional applicable law, and EU-GDPR regulations concerning privacy and data storage. Clinical characteristics for each patient group from which the endomyocardial biopsies were taken can be found in (Table 1). Circulating markers of inflammation and cardiac dysfunction and damage were not significantly different between earlier and later onset toxic cardiomyopathy (Supplementary Figure S1).

**Table 1:** Endomyocardial biopsies for deep RNA sequencing: patient characteristics.

	Control	Early toxicity	Late toxicity
Number (n)	3	6	7
Age (years)	71 ± 5	67 ± 10	67 ± 12
Male/female (ratio)	2:1	1:5	1:6
Weight (kg)	72.0 ± 5.6	67.5 ± 13.2	67.7 ± 14.8
Length (cm)	165.5 ± 2.2	165.6 ± 11.3	166.3 ± 10.5
LVEF (TTE, %)	59 ± 6	27 ± 8	26 ± 8
LVEDD (TTE, mm)	45 ± 3	58 ± 4	51 ± 7
LVESD (TTE, mm)	28 ± 3	47 ± 3	43 ± 9
LVMI (TTE, g/m^2^)	No data	95 ± 11	76 ± 26
NYHA class	I	III	III
Time between first chemo and first signs of heart failure (months)	n/a	29 ± 16	113 ± 86
Concomitant radiotherapy (%)	n/a	50	57
Inflammatory cell count (CD45, n/mm^2)^	None	5.0 ± 1.4	4.4 ± 1.1

### Human material

LV EMB were procured during left heart catheterization. A Mullins sheath (Medtronic^TM^, Switzerland) was introduced in the right or left femoral artery after which LV EMBs were taken by means of a classical biopsy tool (Bipal 7 bioptome, Cordis Corp, Miami Lakes, FL, United States) under fluoroscopic imaging guidance. Biopsies were immediately snap frozen in liquid nitrogen and subsequently stored at −80°C.

### RNA isolation, quantitative RT-PCR

Total RNA was isolated from left ventricular EMBs using the miRVANA isolation kit (Ambion, Warrington, United Kingdom) according to the manufacturer's instructions, without enrichment for small RNAs. Potential genomic DNA contamination was removed using the DNA-free kit (Ambion, Warrington, United Kingdom). To identify and quantify mRNA, cDNA was generated using the BIO-RAD iScript cDNA synthesis kit (#1708891) followed by quantitative real-time PCR using BIO-RAD iQ SYBR Green master mix (#1708882) and a final primer concentration of 200 nM. Primers were ordered from Eurogentec, purified by desalting and sequences can be found in Supplementary Table S1.

### RNA-sequencing

Following total RNA isolation as above, ribosomal RNA (rRNA) was removed using the rRNA Human/Mouse/Rat kit (New England Biolabs, # E6310). Stranded RNA-seq libraries were prepared using the Lexogen SENSE RNA-seq library preparation kit (discontinued). Sequencing was performed at The Babraham Institute, Cambridge, United Kingdom on a HiSeq 2,500 as single end RNA-sequencing. RNA sequencing *fastq* output sequencing files were passed through *fastqc* analysis for basic quality checks and alignment to the reference genome (GRCh38/hg38) was performed using *hisat2* to reference genome (9, 10). Reads were trimmed prior to alignment using TrimGalore, using Phred quality score for base calling cutoff of 20, corresponding to a maximum error of 1 in 100 bases and with a maximum trimming error rate of 0.1 (http://www.bioinformatics.babraham.ac.uk/projects/download.html#trim_galore) (11, 12). Subsequent QC and differential gene expression analysis was performed using SeqMonk v47.0 (The Babraham Institute, United Kingdom. https://www.bioinformatics.babraham.ac.uk/projects/seqmonk/). Differential gene expression analysis was performed using DEseq2 through R, with false discovery rate correction performed using Benjamini-Hochberg correction. Only genes with an adjusted *p* value (*q* value) of less than 0.05 were deemed to be differentially expressed between earlier and later onset cardiotoxic samples (13). RNA-sequencing data are available from the EBI European Nucleotide Archive (ENA) ELIXIR Data Resource (Project # PRJEB51152, Submission #ERA9297316).

### In vitro experiments

Experiments were carried out with H9c2 cells, as described extensively elsewhere (14). The rat embryonic cardiac cell line, H9c2, was purchased from the American Type Culture Collection (ATTC CRL-1446, Rockville, MD, United States). Cells were cultured as reported previously (15) and treated at a confluency of 70%–80%. The complete culture medium (10% FBS in Dulbecco's modified Eagle's medium (DMEM)) was replaced with one with 0.5% FBS, added 1 h before starting experimental treatments, which were also carried out in 0.5% FBS. Cells were exposed to 0.1 µmol/l doxorubicin (in DMSO from Adriblastina, Pfizer, United States) for only 3 h. Cells were then maintained in complete medium for 7, 14 or 21 days before harvesting and passaged each time when a confluency of 70%–80% was reached. Cells and medium were harvested at different time points and immediately stored in TRIzol^TM^ (Ambion, #15596026).

### Mass spectrometry

Genomic DNA was isolated from EMB and H9c2 cells using the Zymo Quick-DNA mi-prep kit (#D3025), according to the manufacturer's instructions. 1 *μ*g of genomic DNA was submitted for liquid chromatography triple quadrupole (LC-QQQ) mass spectrometry (Thermo Scientific) for analysis of DNA cytosine, methylcytosine (MeC) and hydroxymethylcytosine (hMeC) at the Leuven VIB Metabolomics core facility (https://vib.be/labs/vib-metabolomics-core-leuven) as previously described (16). Peak areas for the fragment ions were quantified by external calibration relative to relevant standards.

### Immunoblotting

Total protein lysates were prepared by resuspending H9c2 cell frozen cell pellets in 500 *μ*l RIPA lysis buffer (50 mM Tris HCL pH 8.0, 150 mM NaCl, 1% NP40, 0.5% Na-Deoxycholate, 0.1% SDS) with 2 × Halt™ Protease and Phosphatase Inhibitor Cocktail (Pierce Thermo Scientific #78442). Cells were lysed by incubating for 30 min on ice and then homogenized in a bullet blender for 5 min at setting #9 at 4°C. Protein concentration was measured using a standard BCA Protein assay kit (Pierce™ BCA Protein Assay Kit #23225). 30 *µ*g protein was loaded with 1 × Laemmeli sample buffer (50 mM Tris pH 6.8, 2% SDS w/v, 10% glycerol v/v, 5% *β*-mercaptoethanol v/v and bromophenol blue onto 4%–15% pre-cast Criterion TGX polyacrylamide gels (BIO-RAD #5671084) and run at 200 V. Transfer was then performed onto 0.45 um nitrocellulose membrane (BIO-RAD #1620115) for 2 h at 500 mA. Membranes were then blocked in 5% non-fat milk powder in 1 × TBS-Tween (20 mM Tris, 150 mM NaCl, 0.1% Tween 20). TET2, DNMT3A and GAPDH were probed for using primary antibodies anti-Tet2 (Protein tech # 21207–1-AP). anti-DNMT3A (Protein tech # 20954-1-AP) and anti-GAPDH (Thermo Fischer Scientific # AM4300) respectively in 2.5% bovine serum albumin (Fischer # AK8905-0100) in 1 × TBS-Tween overnight at 4°C at a final concentration of 1:1000. Washes were performed thrice for 10 min in 1 × TBS-Tween followed by secondary antibody incubation with Horse Radish Peroxidase-conjugated anti-rabbit or anti-mouse antibodies (Southern Biotech #OB4050-05 and #OB1031-05) at a final concentration of 1:2000 in 5% non-fat milk in 1 × TBS-Tween. Final washes were performed in 1 × TBS-Tween thrice for 10 min prior to imaging on an Odyssey® XF digital imaging system (LICOR) using the chemiluminescence program for 30 s for each membrane.

### Statistics

Data represent mean ± SEM unless otherwise stated. Statistical significance was calculated by ANOVA with Tukey's *post hoc* multiple testing using Bonferroni correction. For comparisons between two groups, a standard Student's *t*–test was used for normally distributed data, and a Mann-Whitney test was used for non-normally distributed data. Normality testing was performed with the Kolmogorov-Smirnov and Shapiro-Wilk normality test. Data were analyzed statistically using GraphPad Prism v7.0. A two-sided *p* value of < 0.05 was considered as statistically significant. Statistical analysis of RNA-sequencing data is described in the RNA-sequencing section.

## Results

### Early vs. late cardiotoxicity exhibit distinct cardiac transcriptomes

**Figure 1:**
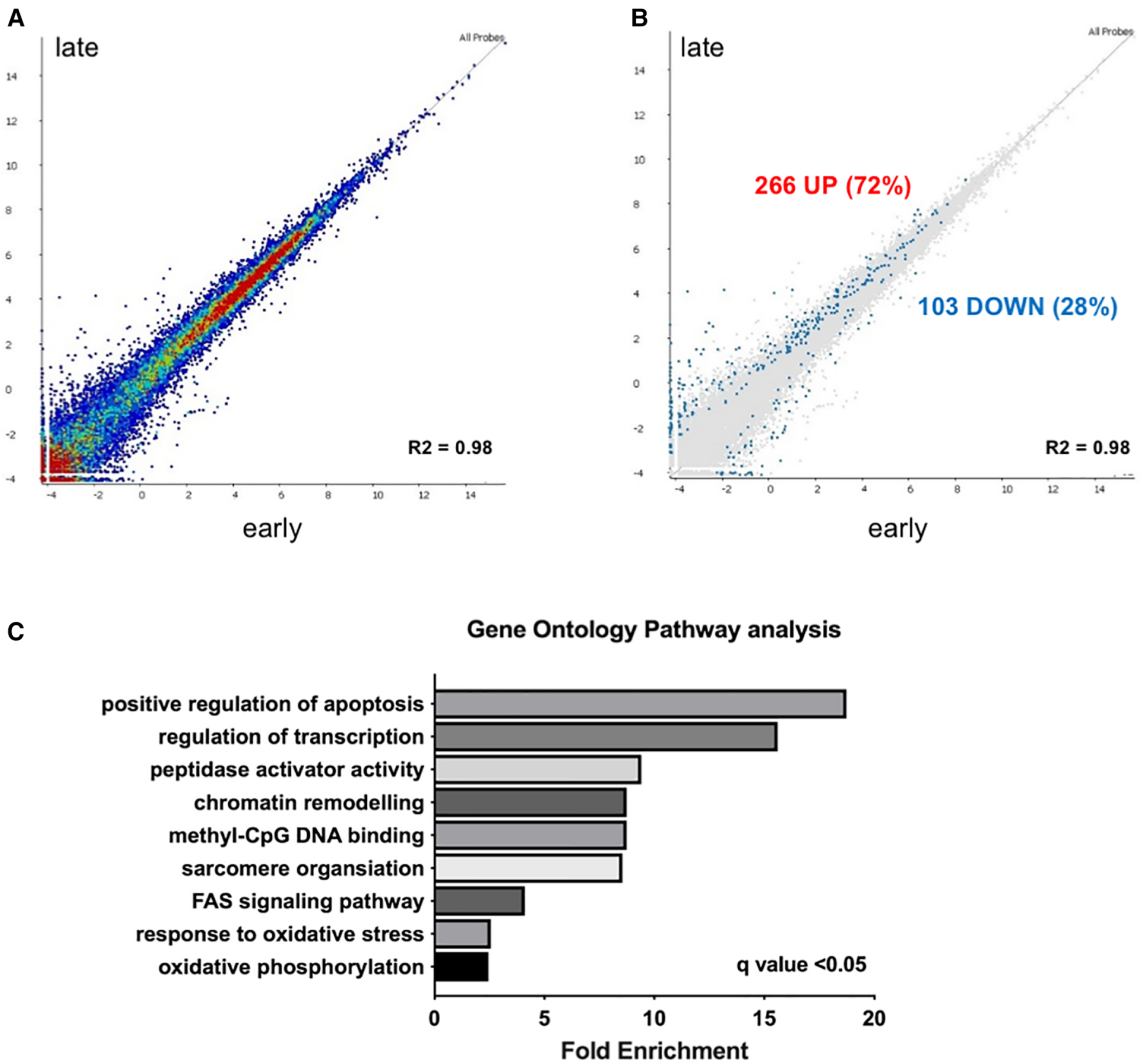
Transcriptome analysis reveals upregulation of epigenetic modifiers in early vs. late chemotherapy-induced heart failure in myocardial patient biopsies. (**A**) relative upregulation of genes in early vs. late chemotherapy-induced heart failure endomyocardial biopsies; (**B**) in total 266 genes (72%) were upregulated. Relative downregulation of genes in early vs. late chemotherapy-induced heart failure endomyocardial biopsies, in total 103 genes (28%) were downregulated; (**C**) gene ontology analysis shows the pathways that are preferentially enriched in early vs. late cardiotoxicity.

Deep RNA sequencing of EMBs from patients with earlier (less than 5 years after the start of chemotherapy) vs. later cardiotoxicity (later than 5 years after the start of chemotherapy) was performed (Figure 1). Correlation of expression of annotated genes between late and early onset cardiotoxicity revealed a coefficient of determination (R squared, *R*^2^) value of 0.98 (Figure 1A), with differential gene expression analysis (DESeq2 (13)) demonstrating a total of 369 differentially expressed genes (DEGs, FDR < 0.05) (Figure 1B). 72% of DEGs (*n* = 266) were upregulated in late as compared with early onset cardiotoxicity (Figure 1B), whereas 28% of genes (*n* = 103) were downregulated (Figure 1B). Gene ontology analysis showed significant enrichment of genes involved in oxidative phosphorylation, the oxidative stress response, FAS signaling, sarcomere organization, methyl-CpG DNA binding, chromatin remodeling, peptidase activator activity, regulation of transcription and positive regulation of apoptosis, in increasing order (Figure 1C). Due to insufficient availability of control human heart left ventricular tissue, RNA-sequencing was performed in toxic cardiomyopathy EMBs only.

### Mediators of DNA methylation are differentially regulated in myocardial tissue in cardiotoxicity-driven heart failure

**Figure 2:**
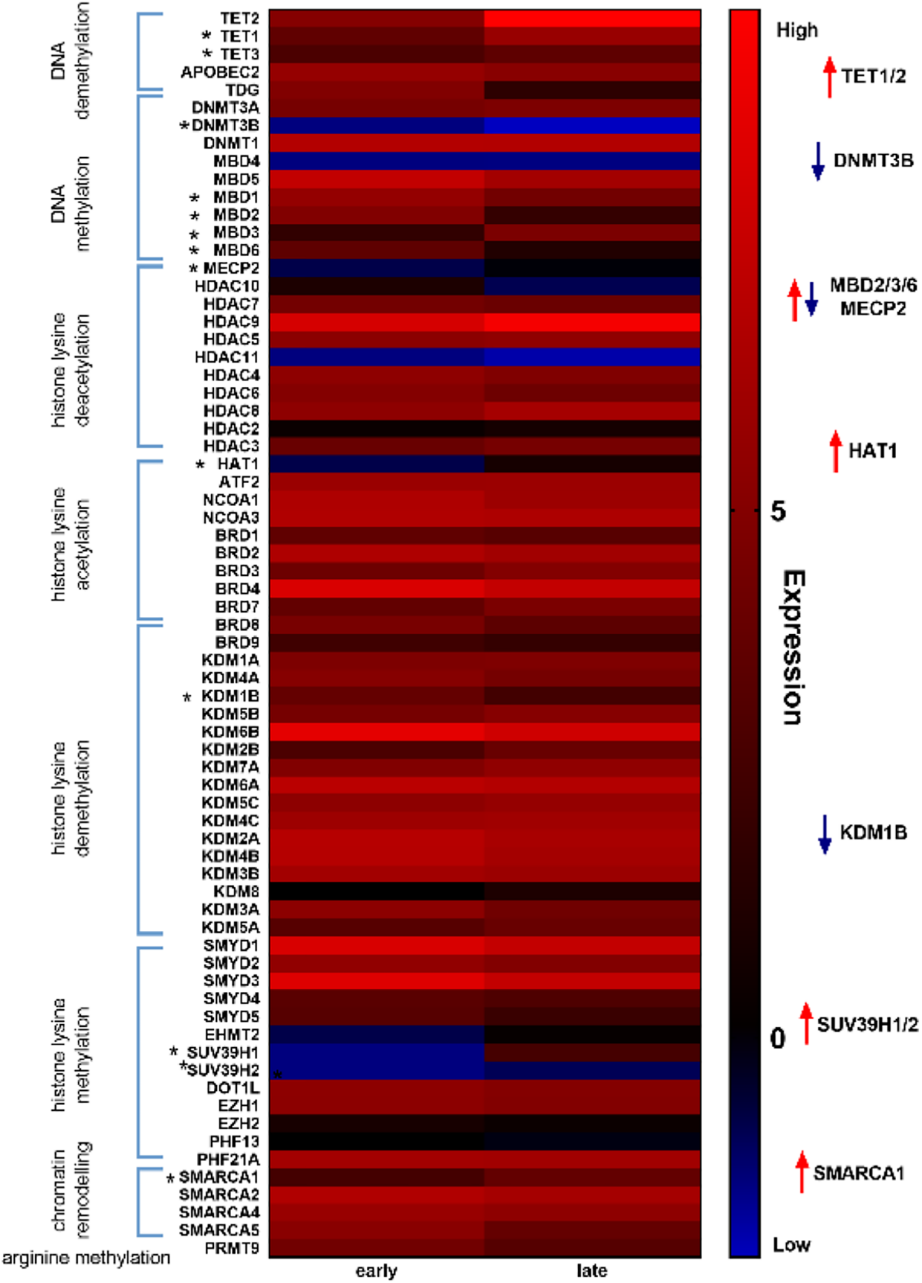
Heat map analysis of epigenetic markers revealed distinct differential regulation of DNA methylation modifiers in early vs. late cardiotoxicity in myocardial patient biopsies. Heat map showing upregulation of genes involved in DNA demethylation (e.g., TET1/2) and downregulation of genes involved in DNA methylation (e.g., DNMT3B).

**Figure 3:**
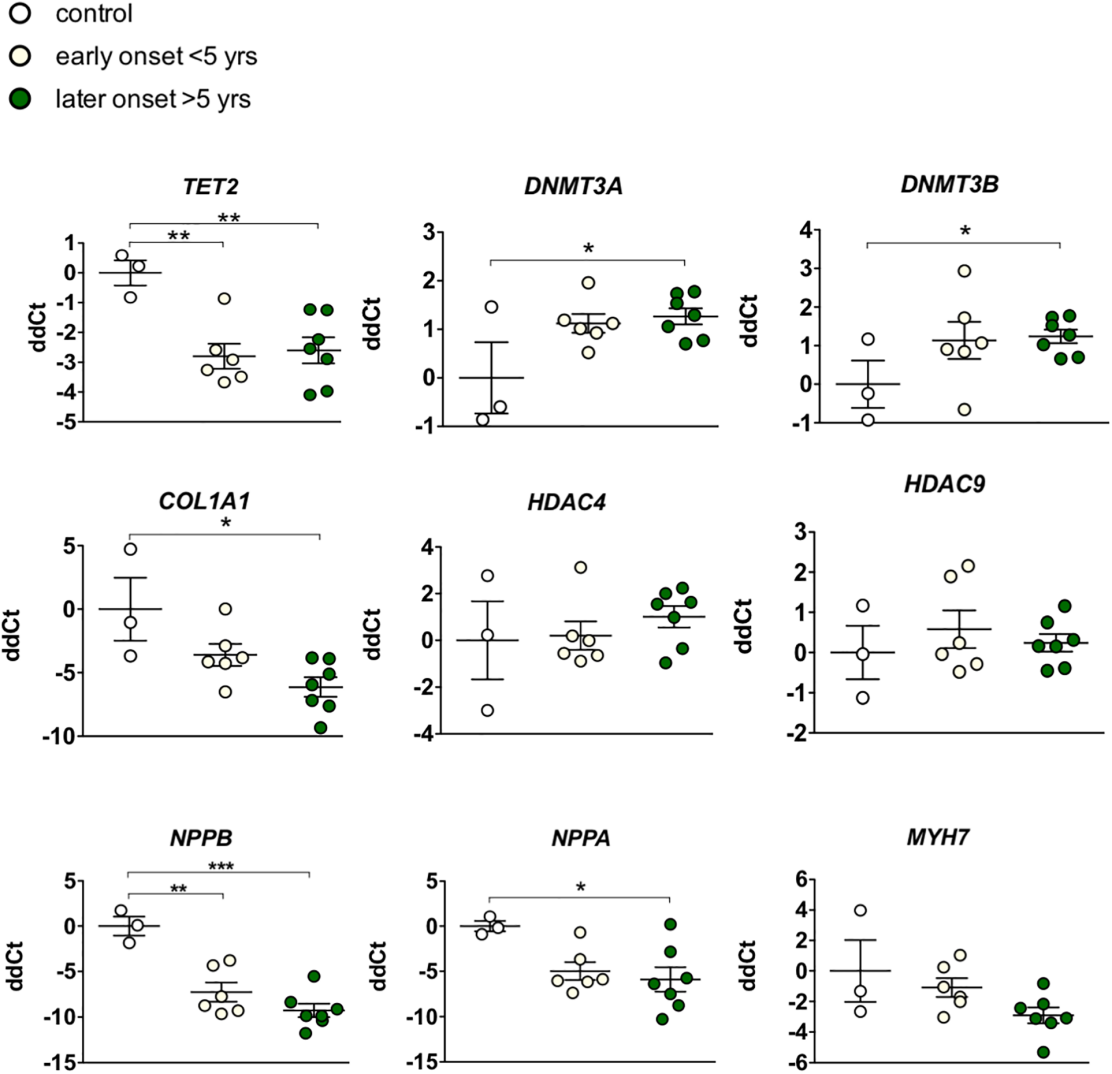
Targeted gene expression analysis of epigenetic markers revealed distinct differential regulation of DNA methylation modifiers in early vs. late cardiotoxicity in myocardial patient biopsies. Confirmation of the upregulation of TET2, and the downregulation of DNMT3A and DNMTB in biopsies of chemotherapy-induced cardiomyopathy patients vs. controls. While COL1A1 was also upregulated in late cardiotoxicity patients, HDAC4/9 and MYH7 were not significantly affected. As expected in heart failure biopsies, NPPA and NPPB genes were upregulated. * Means*p* < 0.05 in one-way ANOVA.

**Figure 4:**
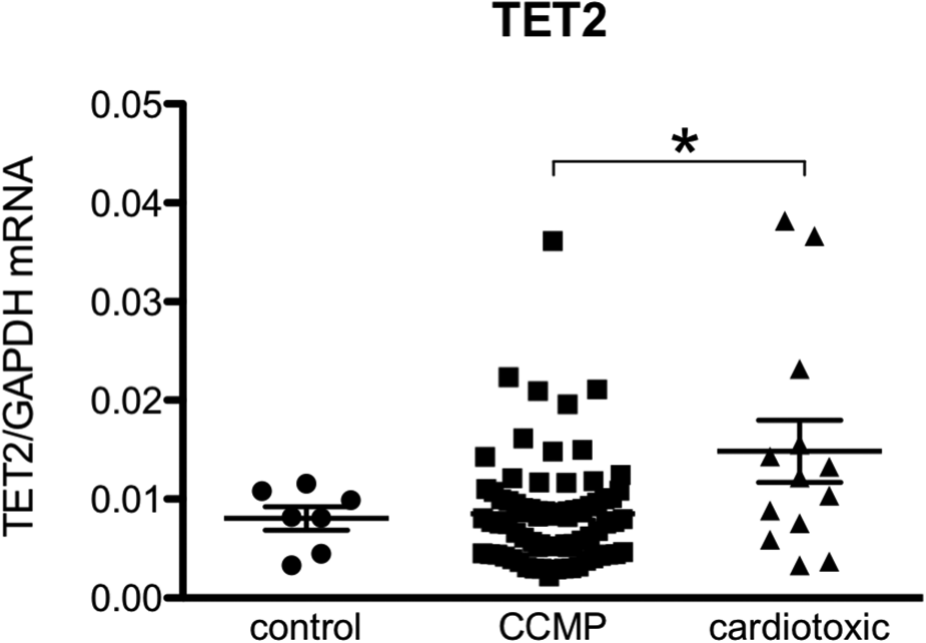
TET2 levels are more elevated in the hearts of patients with anthracycline-induced heart failure compared to non-ischemic non-cardiotoxic cardiomyopathy counterparts. Control patients are patients who underwent CABG but had no structural heart disease and normal left ventricular function (biopsy was procured upon cardiac surgery). CCMP patients are patients with non-ischemic idiopathic cardiomyopathy in whom cardiotoxicity and structural disease e.g., amyloidosis was excluded. Cardiotoxic patients are patient in which a clear link between heart failure and the previous use of anthracycline-based chemotherapy was demonstrated. *Means *p* < 0.05 in one-way ANOVA.

**Figure 5:**
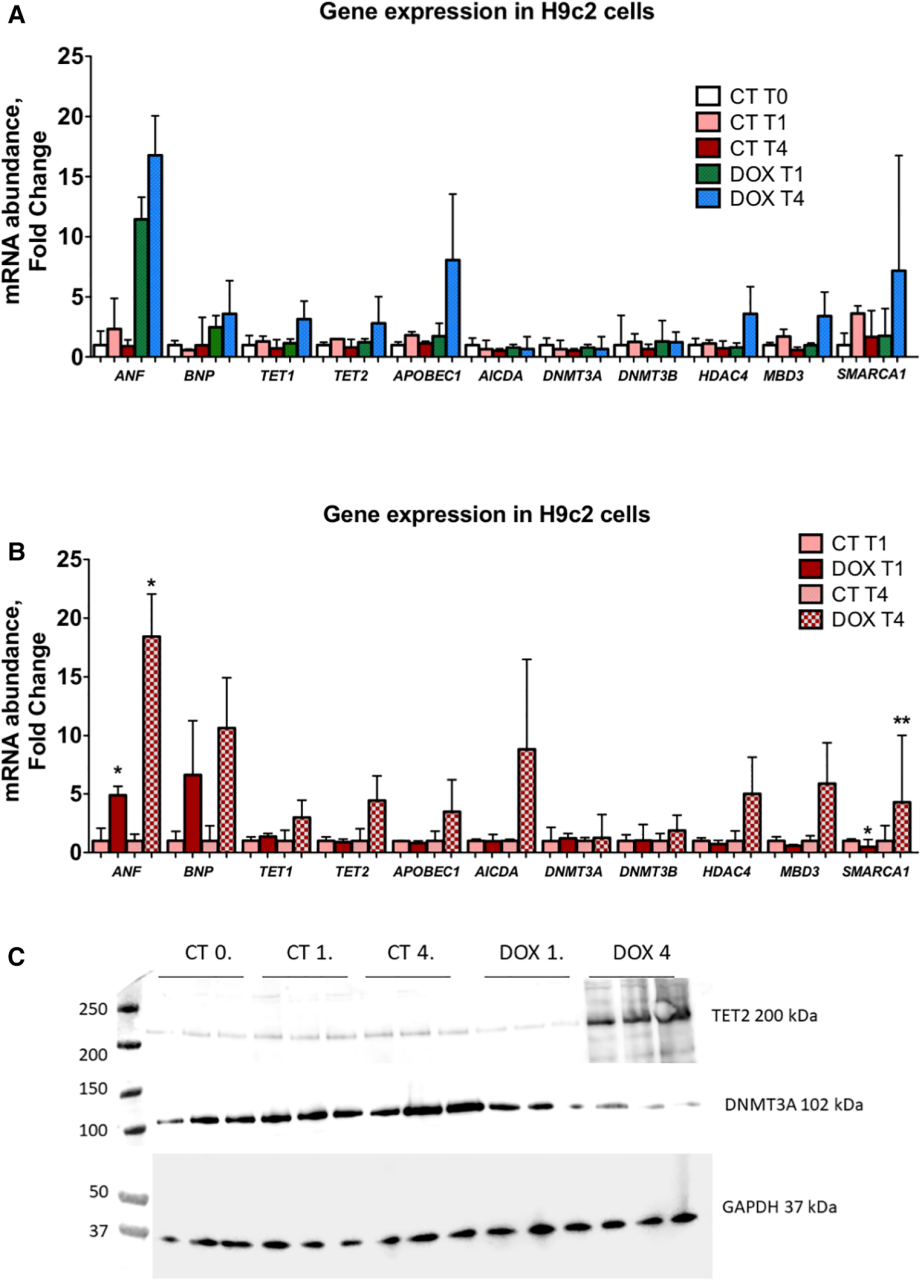
Expression of epigenetic markers is markedly increased in H92c cells 1 and 3 weeks after doxorubicin administration indicative of an epigenetic memory. (**A**) In this panel, mRNA expression data are shown vs. control cells (CT T0). A clear upregulation after passaging cells for one and three weeks after only short doxorubicin administration is observed for ANF, BNP, APOBEC1, TET1, TET2, HDAC4, MBD3 and SMARCA1. DNMT3A and DNMT3B are not significantly altered; (**B**) in this panel, a relative comparison is made between the control and doxorubicin-treated cells at similar time points. Here it is clear that the abovementioned epigenetic regulators are significantly upregulated and that this upregulation is more pronounced the longer cells are passaged, indicative of a memory effect. *p < 0.05 in one-way ANOVA. Abbreviations: CT = control; T0 = no doxorubicin; T1 = 1 week time point; T4 = 4 week time point; DOX = doxorubicin. (**C**) Tet2 and Dnmt3a protein expression in H9C2 cells.

Given our hypothesis that epigenetic mechanisms play a role in late onset and long term cardiotoxic effects of anthracycline treatment along with methyl CpG DNA binding being a differentially expressed pathway in the deep sequencing analysis, we focused on expression of epigenetic readers, writers and erasers in EMBs (Figure 2). Heat map clustering showed important upregulation of genes involved in active DNA demethylation, including TET1/2, and downregulation of *de novo* DNA methyltransferase DNMT3B (Figure 2). Differential mRNA expression of genes involved in DNA methylation metabolism was confirmed by performing classical RT-qPCR in EMBs (Figure 3). Moreover, in a larger biopsy cohort, it was shown that Tet2 expression was more abundantly expressed in cardiotoxicity biopsies vs. control biopsies, but also compared with non-cardiotoxic non-ischemic cardiomyopathy patients (Figure 4). For a sub-set of genes identified as differentially expressed from the RNA-sequencing data, methylated or hydroxymethylated DNA immunoprecipitation qPCR ((h)MeDIP-qPCR) experiments were performed at within the gene promoter regions (−1000–0 bp from TSS) (Figures 5A-B). Importantly, these findings are unlikely due to a more cardiac phenotype in later onset cardiotoxicity, as measures of cardiac dysfunction, such as LV ejection fraction (LVEF), end diastolic LV dimensions (LVEDD), and myocardial inflammatory cell infiltration did not differ significantly between earlier and later cardiotoxicity (see Table 1).

### Short-term exposure to doxorubicin leads to long-lasting alterations in epigenetic modifiers and modifications in cardiomyocytes

**Figure 6:**
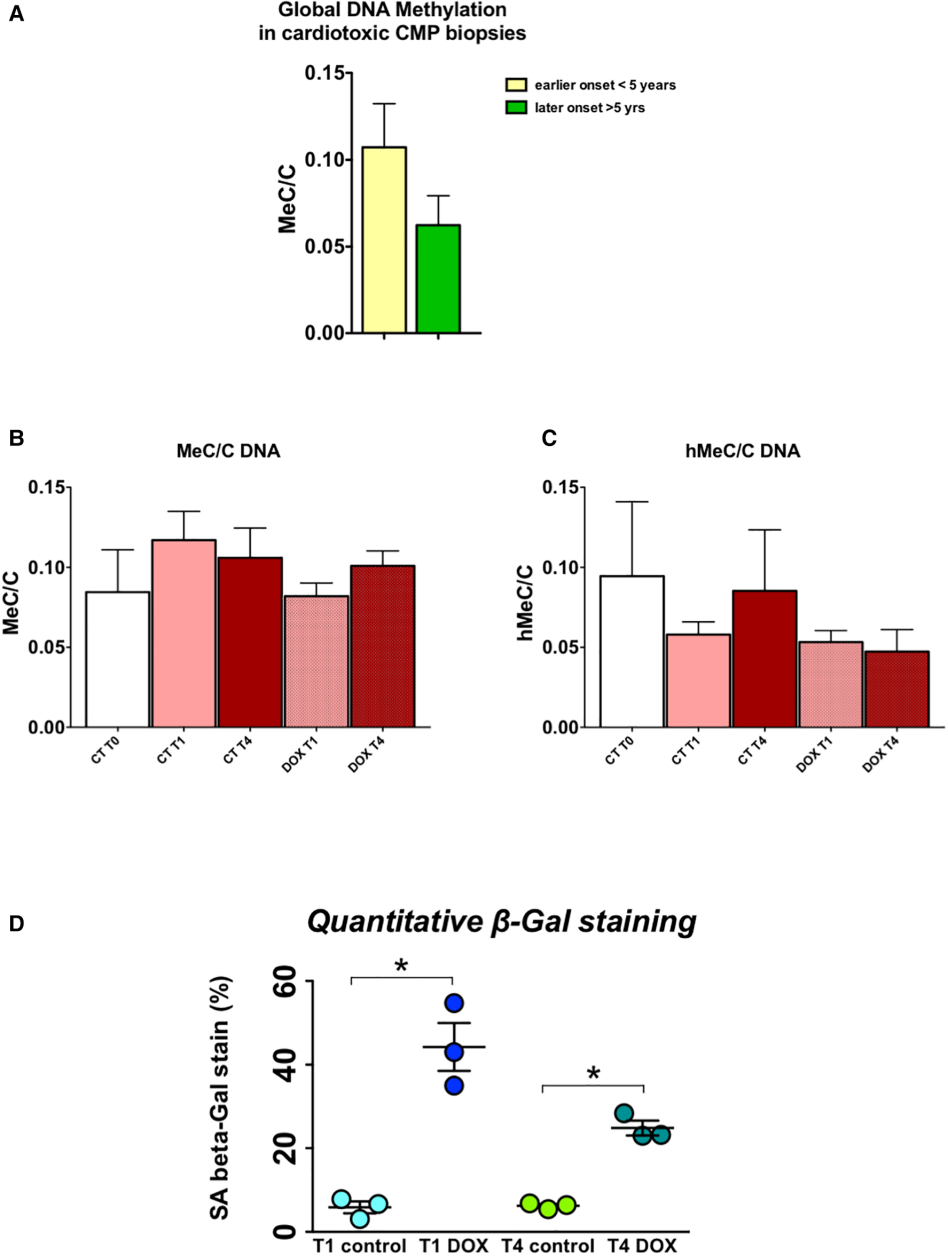
DNA methylation/demethylation patterns in biopsies of patients with chemotherapy-induced heart failure mimic patterns observed in H9c2 cells, which show increased senescence even long after a short-term doxorubicin administration. (**A**) Relative global levels of methylated cytosine vs. demethylated cytosine levels (MeC/C) in early vs. late cardiotoxicity biopsies shows increased methylation at a later time point after administration of chemotherapy, measured by liquid chromatography mass spectrometry; (**B**) Relative MeC/C levels trend to decrease over time after doxorubicin administration in H9c2 cells, while (**C**) hMeC/C levels are also lower; *p < 0.05 in two-way ANOVA. (**D**) Quantitative beta galactosidase staining – a marker of cell senescence – shows strongly increased senescence in doxorubicin cells, one and three weeks after treatment, indicative of a memory effect.

**Figure 7:**
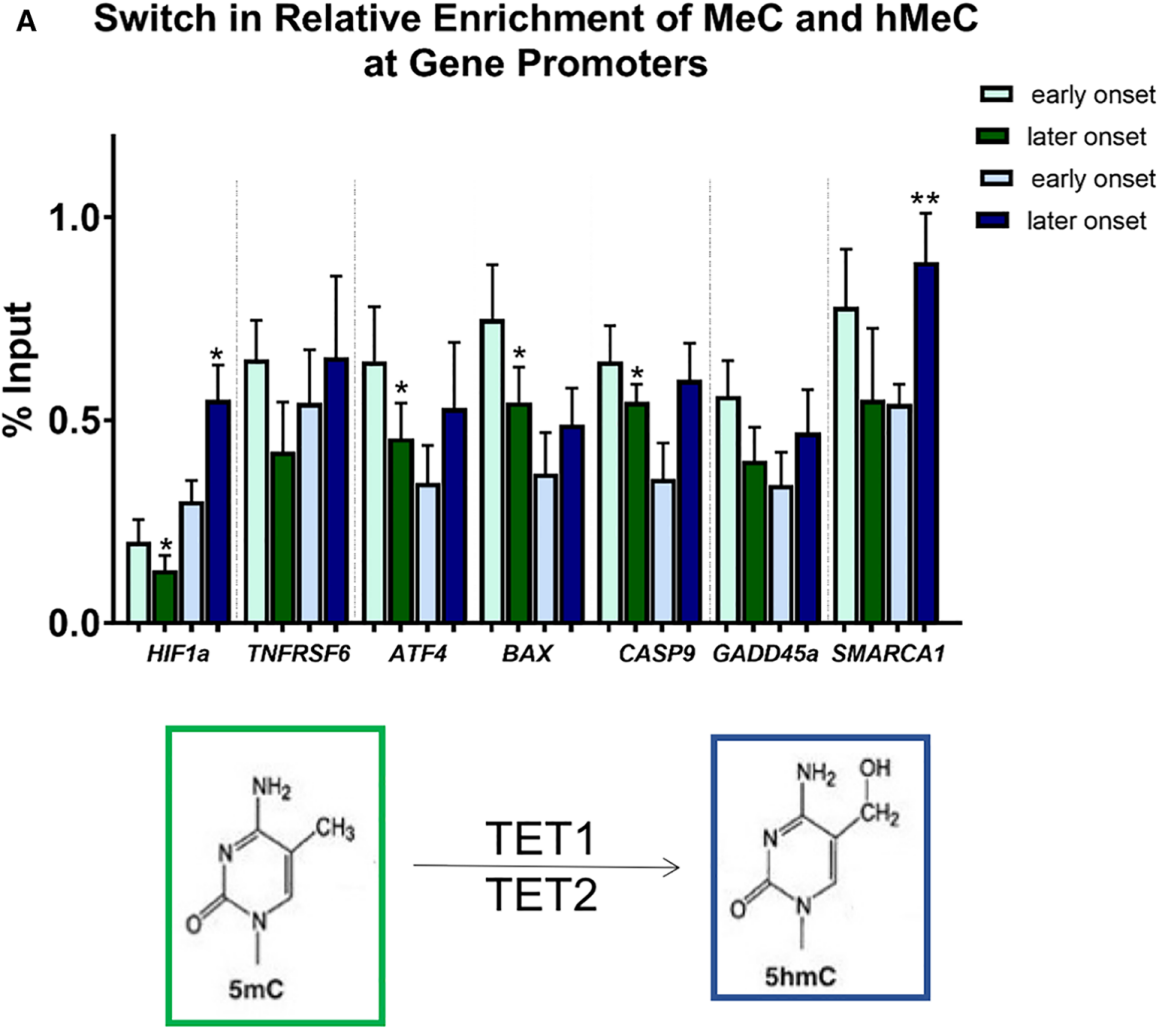
Differential methylation and demethylation is associated with changes in expression patterns of genes responsible for epigenetic regulation. (**A**) Chromatin immunoprecipitation analysis revealed a loss of DNA methylation and an increase in hydroxymethylation at promotors of selected differentially expressed genes in later vs. earlier onset toxic cardiomyopathy. *p < 0.05, **p < 0.01.

H9c2 cardiomyocytes in culture were treated for three hours with a concentration of doxorubicin known to cause important intracellular changes along with expression of senescence markers, but no cell death. The hallmark of this type of toxicity is the accumulation of SA-*β*-galactosidase (14, 15, 17). Following doxorubicin treatment, the medium was washed away and substituted with fresh culture medium. Subsequently, cells were cultured and passaged once they reached a confluency of 70%–80%. No other treatments were performed. The expected increase in SA-*β*-galactosidase levels persisted up to three weeks after exposure to doxorubicin (Figure 6D). Furthermore, when comparing untreated cells to doxorubicin-treated cells, even and especially three weeks after short-term treatment, *Nppa, Nppb, Tet/2* and other genes involved in active DNA demethylation were markedly upregulated (Figures 5A-C). Whilst *de novo* methyltransferases Dnmt3a and Dnmt3b were not significantly changed at the mRNA level, Dnmt3a was downregulated at the protein level in H9c2 cells treated with doxorubicin compared with control cells and more so following three weeks than one week of treatment (Figures 5A-C). Accordingly, and suggesting a functional consequence of these gene expression changes, global DNA methylation levels were decreased in later vs. earlier cardiotoxicity biopsies (Figure 6A). When the three-week vs. one-week time point were compared, epigenetic modifier alterations were still persistent and even more pronounced (Figures 5B-C). These alterations coincided with a loss of DNA methylation and a gain in hydroxymethylation (Figures 6B-C)*,* reflecting the epigenetic changes seen in the endomyocardial biopsies (Figure 6A). The differential methylation and demethylation were associated with changes in expression patterns of epigenetic regulators in the EMBs (Figure 7).

## Discussion, conclusion, clinical implications

**Figure 8:**
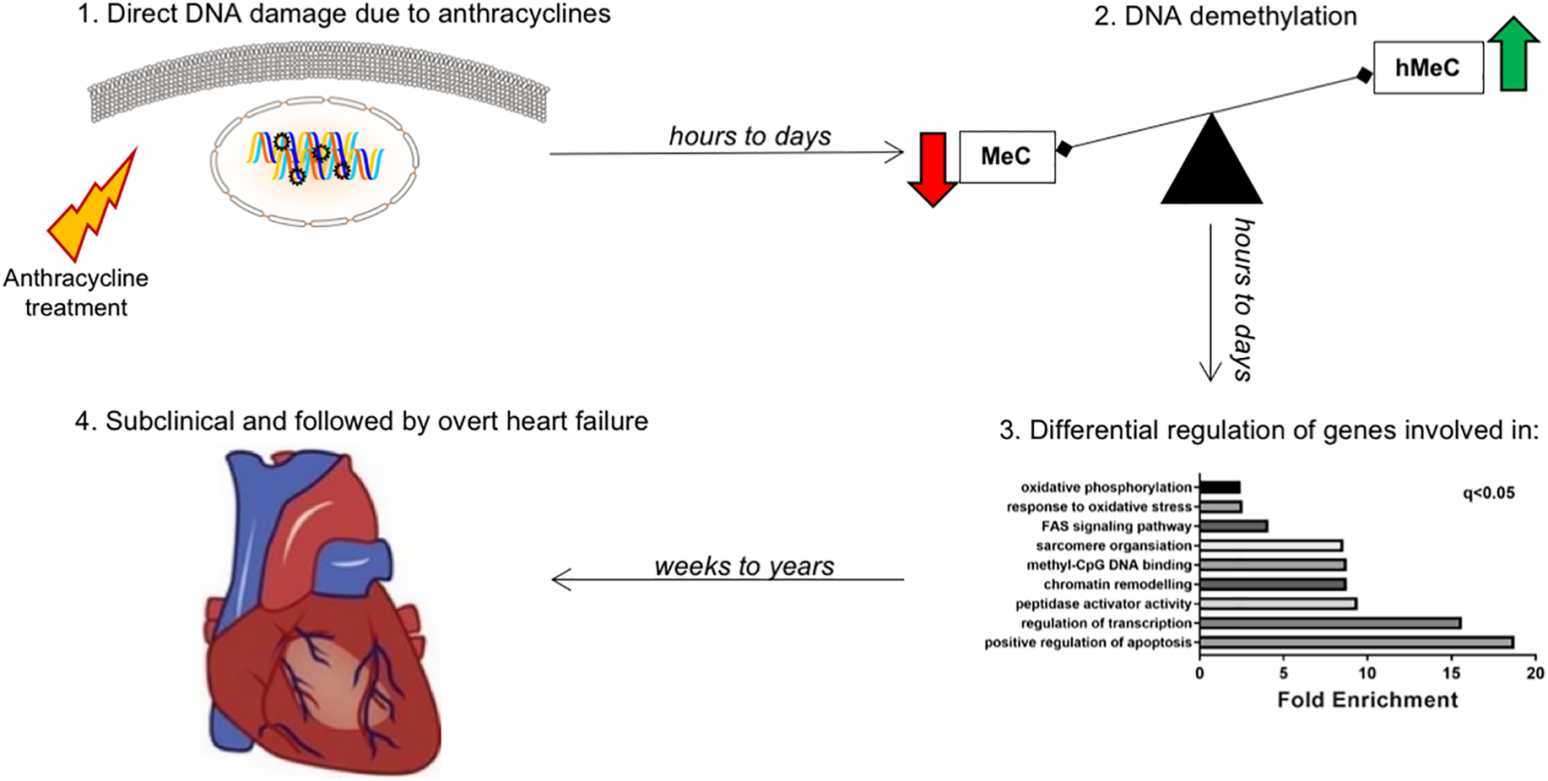
Working model: late-onset chemotherapy-associated heart failure is induced and maintained by a “epigenetic memory” from anthracycline administration. Hypothetical mechanism explaining the effect of cardiotoxicity even years after short-lived chemotherapy administration. The effect of anthracycline is an acute and long-lasting deregulation of genes involved in epigenetic modifications, especially DNA methylation/demethylation. While methylation of genes (*via* cytosine) leads to transcriptional repression, hydroxymethylation leads to transcriptional induction. This hMeC/MeC balance shift leads to increased senescence of cardiomyocytes, ultimately contributing to long term anthracycline cardiomyopathy.

We found major changes in the gene expression profile of the hearts of patients who suffered from anthracycline-related cardiomyopathy within or after 5 years from treatment. In particular, we highlighted differences in the expression of genes involved in the regulation of DNA methylation, which were associated with a loss of DNA methylation and a gain in DNA hydroxymethylation in EMBs of late- vs. early-onset anthracycline cardiomyopathy. Moreover, the same, persisting epigenetic modifications were observed in a cell model of doxorubicin cardiotoxicity. Overall, these data indicate that anthracyclines cause long-term changes in the cardiac transcriptome and epigenome, possibly contributing to the development of cardiomyopathy and HF (Figure 8).

### Is there evidence of a pivotal role for epigenetic memory in other contexts?

Cells, organs and organisms must respond to changes in their environment to adapt and survive. Epigenetic memory plays a crucial role in these alterations. Three types of epigenetic memory are generally distinguished: cellular memory, transcriptional memory and transgenerational memory (18). The latter concept is held responsible for e.g., altered human physical traits in several generations post the historical Dutch famine crisis (19–21). Also, the placenta is involved in early growth and has been linked to several diseases that develop in later late, notably cardiovascular diseases (22). In immunology, especially memory T-cells retain a long-term epigenetic imprint that confers constitutive and inducible gene expression associated with a rapid recall response capacity (23). In the last decade, emerging evidence pointed out an important role for epigenetic memory in different aspects of cardiovascular disease e.g., atherosclerosis (24), post-myocardial infarction remodeling and HF (25). For example, genome-wide DNA methylation profiling of atherosclerotic vs. normal human aorta revealed altered global DNA hypermethylation status, and the identified locations were mapped to genes known to be involved in atherosclerosis (26). A multitude of studies with small sample sizes has been carried out to investigate DNA methylation in HF. Interestingly, it was even investigated whether ablation of Dnmt3a and 3b in genetically manipulated mice did affect the phenotype response to pressure overload, which was not the case. However, transcriptional responses altered substantially in these models (27, 28). We recognize the limitation of using human EMB samples in our study, once cardiac dysfunction has already ensued and progress in developing appropriate pre-clinical models of anthracycline-associated CMP needs to be made. Although some evidence is certainly correlative, a lot of these studies are hypothesis-generating and feed the idea that epigenetic memory must be of importance in HF.

### Contribution of other cardiac cell types in the pathophysiology of toxic cardiomyopathy

Whilst we modeled acute and long-term toxic cardiomyopathy in a cardiomyocyte cell line in this study, we acknowledge that they are outnumbered by other cells types in the heart 1:3. Other cell types have been shown to play a role in the pathophysiology of acute and late-onset cardiotoxicity. Endothelial cells and fibroblasts comprise the major cell types in the heart by number (29).

There is pre-clinical evidence for fibroblast activation, apoptosis and senescence due to exposure to anthracyclines as well as increased myocardial strain in survivors of childhood cancers (30–32). These data provide evidence of fibroblast dysfunction in cardiotoxicity.

A further abundant cell type in the mammalian heart is the endothelial cell. Endothelial dysfunction is a major contributor to cardiac and vascular disease and systolic and diastolic dysfunction (33). Anthracycline exposure has been linked to changes in endothelial cell biology. In human endothelial cells exposure to anthracyclines in culture, nuclear damage and apoptosis was observed (34). Chemotherapy treatment has been linked with disordered VEGF signaling in human endothelial cells, which could affect angiogenesis *in vivo* (35).

A further key mechanism underlying cardiac damage in response to cardiotoxic drugs is that of DNA damage driven by reactive oxygen species (ROS). The heart muscle and cardiomyocytes in particular are prone to ROS damage due to their high mitochondrial content and energy turnover. Anthracycline drugs can also enter the nucleus to cause direct DNA damage (34). Ensuing molecular and cellular dysfunction as a result of increased elevated ROS includes DNA damage, somatic mutation and nuclear damage, mitochondrial dysfunction, altered calcium handling and reduced protein synthesis and cardiac protein disarray including sarcomeric proteins (36). These changes can lead to acute and chronic pathological changes in cardiomyocyte function and death.

A key molecular function of doxorubicin is inhibition of topoisomerase II (TopII) and evidence shows that TopII is depleted in cardiomyocytes of mice exposed to doxorubicin (37). TopII helps to maintains higher order DNA structure and organization. Depletion of TopII can lead to nuclear disarray in cardiomyocytes, contributing to cardiomyocyte death as an acute response to anthracycline treatment, increasing risk of heart failure later.

We acknowledge that through the aforementioned mechanisms, apoptosis of cardiac cells as part of the acute response to chemotherapy exposure, contributes to a decline in cardiac function. Our data, however, uniquely support long-term induction of pro-apoptotic gene expression in the heart, more than half a decade post chemotherapy cessation, that is associated with loss of promoter methylation at these gene loci.

Overall, with cardiomyocytes being the only terminally-differentiated cell type in the heart, long-lasting cellular and molecular effects, including epigenetic changes, in the heart post chemotherapy cessation are most likely to derive from the cardiomyocytes, contributing to long-term cardiac dysfunction.

### What is the potential for epigenetic drugs in the treatment of anthracycline-associated cardiotoxicity?

If epigenetics plays a key role in anthracycline-induced cardiotoxicity, can these processes be therapeutically targeted? Inhibitors of different HDACs are being investigated in numerous clinical trials on metastatic cancers e.g., multiple myeloma, melanoma, gastric cancer, and so on. No such trials are currently being performed in HF patients let al.one cardiotoxic HF patients. As far as DNMT is considered, different products are used for the treatment of hematologic malignancies and solid tissue tumors (e.g., 5-azacytidine and decitabine being DNMT inhibitors), hence they might dramatically influence the epigenome by their direct action on DNMT in non-tumor cells. These off-target effects (in non-tumor cells) might be responsible for the epigenetic alterations we observed in our study. However, inhibiting these pathological effects should not come at the cost of the beneficial effects of chemotherapy on the oncologic process.

One further caveat is the predominant use of human cardiac biopsies from female toxic cardiomyopathy patients. Whilst this means we cannot deduce any sex differences in the data, females outnumber males in clinical cohorts of severe toxic cardiomyopathy (38).

### Future perspectives

Our paper sheds a ray of light on the conundrum of the protracted toxic effect of chemotherapy on the heart. Nevertheless, substantial challenges concerning care for these patients remain. Currently – irrespective of common risk factors for cardiovascular disease e.g., hypercholesterolemia –stratifying low vs. high cardiotoxicity risk remains a challenge for physicians. Also, despite considerable progress in the last two decades, dedicated imaging tools especially for the detection of early cardiac dysfunction are still lacking or insufficiently validated. Finally, more research diving into the mechanism of cardiotoxicity and further development of translationally-relevant pre-clinical models are needed as to develop specific therapeutics to treat this impactful condition.

## Data Availability

The datasets presented in this study can be found in online repositories. The names of the repository/repositories and accession number(s) can be found below: https://www.ebi.ac.uk/ena, ERA9297316.
