# Supplementary material for: Differential expression of epigenetic modifiers in early and late cardiotoxic heart failure reveals DNA methylation as a key regulator of cardiotoxicity
Source: Front Cardiovasc Med. 2023 Mar 9;10:884174. doi: 10.3389/fcvm.2023.884174 (PMC10034031; doi:10.3389/fcvm.2023.884174)
Supplement: Supplementary file 1 [file Table1.pdf]

Supplementary Table 1

Human qPCR primer sequences

NPPA F	CAGACGTAGGCCAAGAGAGG
NPPA R	ACGGCATTGTACATGGGATT
NPPB F	TTCTTGCATCTGGCTTTCCT
NPPB R	GGACTTCCAGACACCTGTGG
MYH7 F	GTGAAAGTGGGCAATGAGT
MYH7 R	TGGTGAAGTTGATGCAGAGC
HDAC4 F	AGGTGAAGCAGGAGCCCATTGA
HDAC4 R	GGTAGTTCCTCAGCTGGTGGAT
COL1A1 F	GTGCTAAAGGTGCCAATGGT
COL1A1 R	ACCAGGTTCACCGCTGTTAC
HDAC9 F	TCTCGTCTCCAGGACTCACTCT
HDAC9 R	GCACTGGTGTTTCAGCATCAAGG
TET2 F	GCTGGAGCACAAGTCACAAA
TET2 R	CACCTGGTGTGAGGGTTTTT
DNMT3A F	GCAGCTACTTCCAGAGCTTCA
DNMT3A R	GCAATGACCTTGGCTTTCTT
DNMT3B F	GCAGGTCCGAACTCGAAATA
DNMT3B R	GGACTCGTCCACATGGTTG
18 S F	AAACCACAGGCAAACACCTC
18 S R	GCACTTTGGGTGGTCAAGTT
CASP9 F	GTTTGAGGACCTTCGACCAGCT
CASP9 R	CAACGTACCAGGAGCCACTCTT
BAX F	TCAGGATGCGTCCACCAAGAAG
BAX R	TGTGTCCACGGCGGCAATCATC

Rat qPCR primer sequences

ANF F	CGTATACAGTGCGGTGTCCAAC
ANF R	CCTCATCTTCTACCGGCATC
BNP F	GGTCTCAAGACAGCGCCTTCC
BNP R	CTTCCTAAAACAACCTCAGCCCGTC
TET1 F	GTCCTGAACTGTCCCCTGAA
TET1 R	GGCATTAAGGGGAGAAAAGC
TET2 F	TTGGACTTCTGTGCTCATGC
TET2 R	TCCTCCTGAGCTTCCACACT
APOBEC1 F	CCAAAACACCAACAAACACG
APOBEC1R	CCAGGACAGGAACCAGGTAA
AICDA F	TGGGCCAATCGTAATAGAGC
AICDA R	CAGAGCTAGGGTGGTTCTGC
DNMT3A F	GGACACCAGCATCTCCTCTC
DNMT3A R	GCTCCTGACGCTCTTCCTTA
DNMT3B F	GGTTAAGCGGCCCAAGTAA
DNMT3B R	ACTCCCGAATCTGGAAAGGA
HDAC4 F	GCATGTTCTCGTGGGAATTT
HDAC4 R	CTCATTGCTAGCAGCGTCAG
MBD3 F	AGAAGAACCCTGGTGTGTGG
MBD3 R	TGTACCAGCTCCTCCTGCTT
SMARCA1 F	CGTGGTCCTAGAGGATGAGC
SMARCA1 R	CTCCTTCTTCTCGCCCTTCT
18 S F	ATCCATTGGAGGGCAAGTC

18 S R	CGCTCCCAAGATCCAACTAC
CASP9 F	ACGTGGACTGTGAGAAGCTT
CASP9 R	AGCCATGAGAGAGGATGACC
BAX F	TCATGAAGACAGGGGCCTTT
BAX R	CTGCAGCTCCATGTTGTTGT

Human ChIP primer sequences

HIF1A F	GGAGAAAGAGAGCAGGAGCA
HIF1A R	CTGAGAAGGGATTTCGGTTG
TNFRSF6 F	GGGCACGTGTCCACTTTTA
TNFRSF6 R	ACTCCCTGTCCCTCAACTCC
ATF4 F	GGAGCCTTGTGACTTTGAGC
ATF4 R	AATAACCACAGGCCATCTGC
BAX F	GCCTCTGAGCTTTTGCACTT
BAX R	CGTCCAATCGCAGCTCTAAT
CASP9 F	GGTGACCCAGAATTGACC
CASP9 R	GCAGGACGCATCTCCAAC
GADD45A F	TTAACTTGGTTCCCTGCCATC
GADD45A R	CCTGATGGCTGGTTTGATTT
SMARCA1 F	TAACTTGGTTCCCTGCCATC
SMARCA1 R	CCTGATGGCTGGTTTGATTT
