# Supplementary figures and images for: Differential expression of epigenetic modifiers in early and late cardiotoxic heart failure reveals DNA methylation as a key regulator of cardiotoxicity
Source: Front Cardiovasc Med. 2023 Mar 9;10:884174. doi: 10.3389/fcvm.2023.884174 (PMC10034031; doi:10.3389/fcvm.2023.884174)

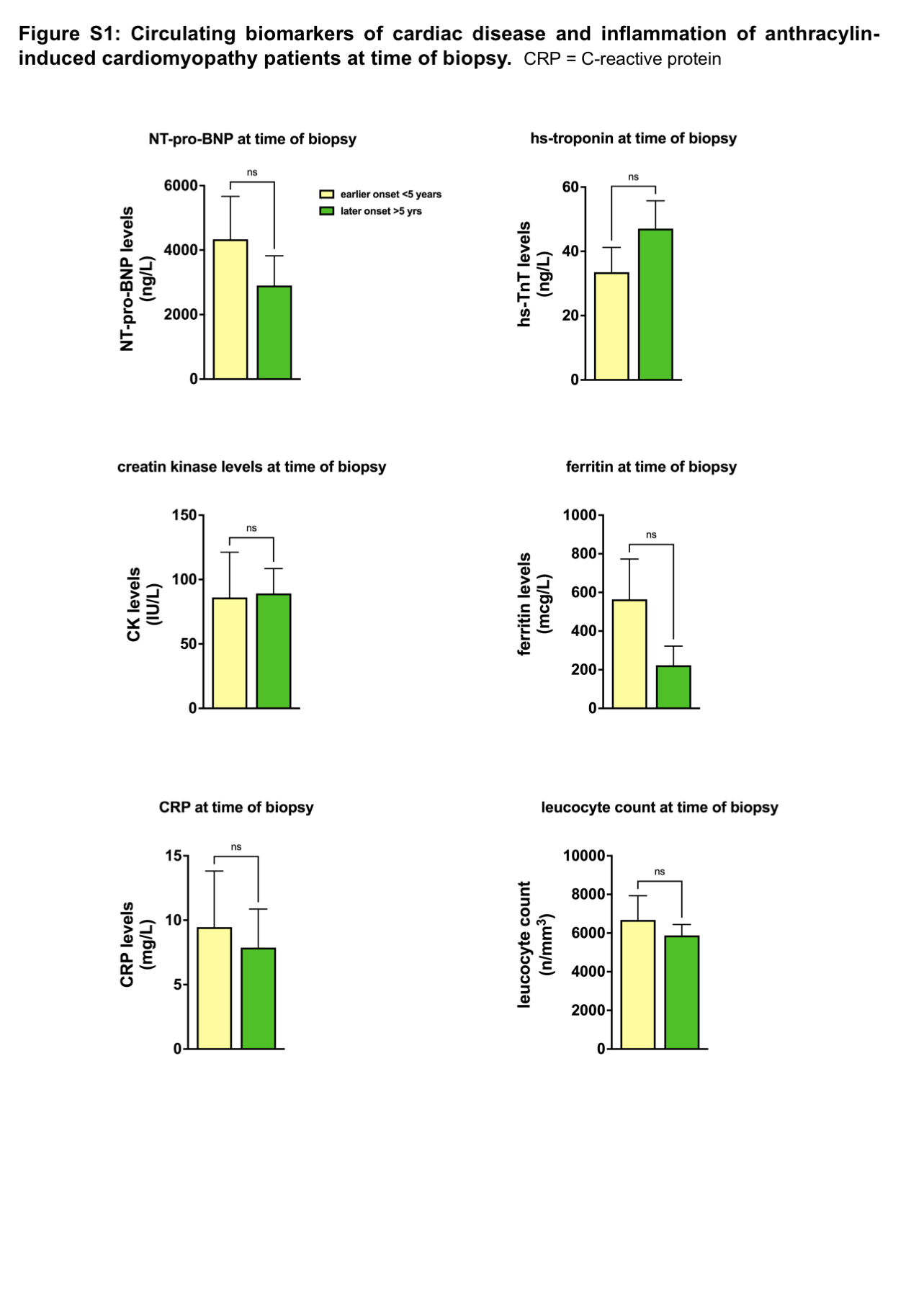

Supplement: Supplementary file 2 [file Image1.tiff]

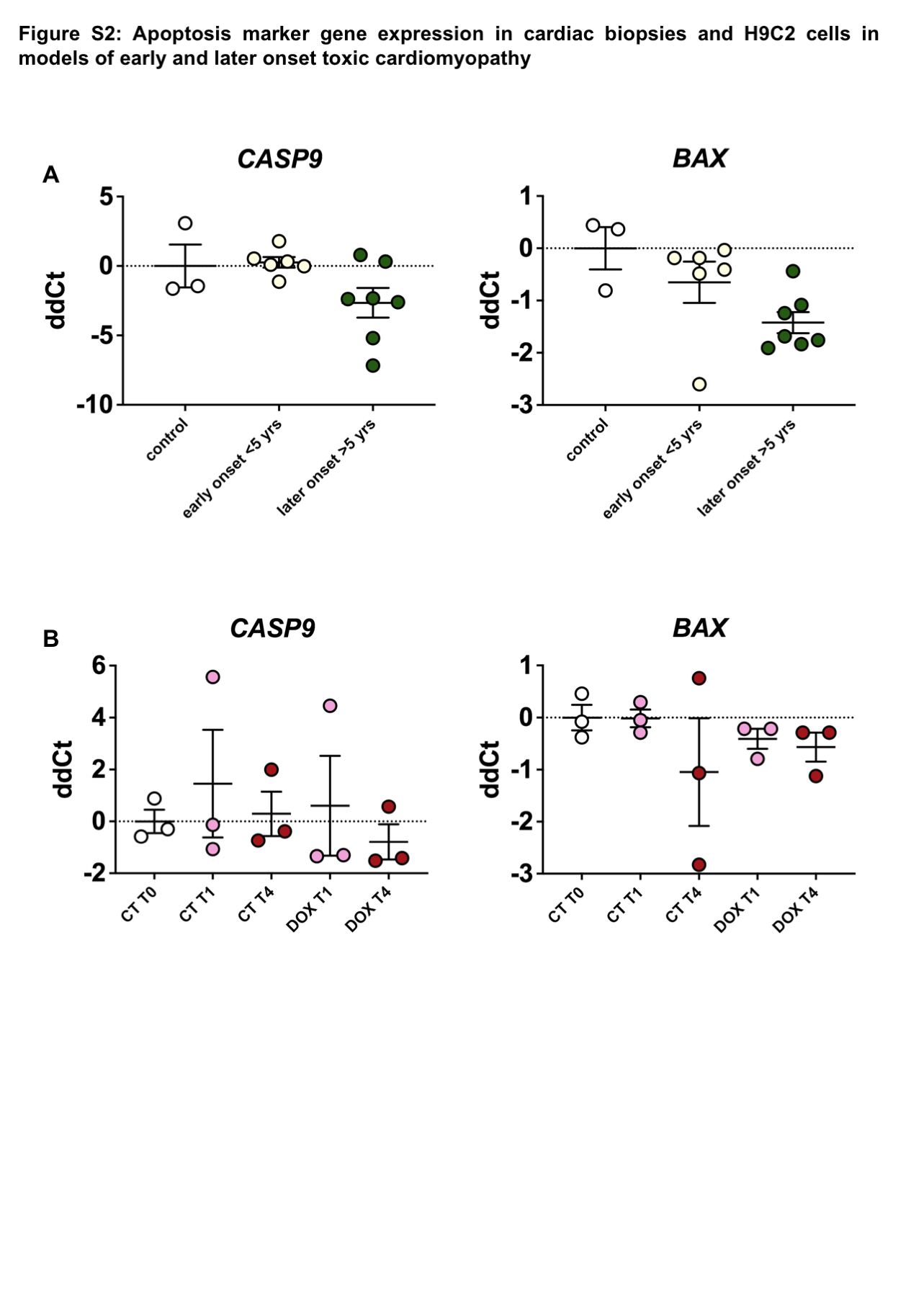

Supplement: Supplementary file 3 [file Image2.tiff]
